# The Molecular Mechanism of Substrate Engagement and Immunosuppressant Inhibition of Calcineurin

**DOI:** 10.1371/journal.pbio.1001492

**Published:** 2013-02-26

**Authors:** Simina Grigoriu, Rachel Bond, Pilar Cossio, Jennifer A. Chen, Nina Ly, Gerhard Hummer, Rebecca Page, Martha S. Cyert, Wolfgang Peti

**Affiliations:** 1 Department of Molecular Pharmacology, Physiology and Biotechnology, Brown University, Providence, Rhode Island, United States of America; 2 Department of Molecular Biology, Cell Biology and Biochemistry, Brown University, Providence, Rhode Island, United States of America; 3 Department of Biology, Stanford University, Stanford, California, United States of America; 4 Laboratory of Chemical Physics, National Institute of Diabetes and Digestive and Kidney Diseases, National Institutes of Health, Bethesda, Maryland, United States of America; 5 Department of Chemistry, Brown University, Providence, Rhode Island, United States of America; Brandeis University, United States of America

## Abstract

Ser/thr phosphatases dephosphorylate their targets with high specificity, yet the structural and sequence determinants of phosphosite recognition are poorly understood. Calcineurin (CN) is a conserved Ca^2+^/calmodulin-dependent ser/thr phosphatase and the target of immunosuppressants, FK506 and cyclosporin A (CSA). To investigate CN substrate recognition we used X-ray crystallography, biochemistry, modeling, and in vivo experiments to study A238L, a viral protein inhibitor of CN. We show that A238L competitively inhibits CN by occupying a critical substrate recognition site, while leaving the catalytic center fully accessible. Critically, the 1.7 Å structure of the A238L-CN complex reveals how CN recognizes residues in A238L that are analogous to a substrate motif, “LxVP.” The structure enabled modeling of a peptide substrate bound to CN, which predicts substrate interactions beyond the catalytic center. Finally, this study establishes that “LxVP” sequences and immunosuppressants bind to the identical site on CN. Thus, FK506, CSA, and A238L all prevent “LxVP”-mediated substrate recognition by CN, highlighting the importance of this interaction for substrate dephosphorylation. Collectively, this work presents the first integrated structural model for substrate selection and dephosphorylation by CN and lays the groundwork for structure-based development of new CN inhibitors.

## Abstract 2

Structural analyses show that a viral protein and immunosuppressant drugs inhibit the phosphatase calcineurin by preventing substrate binding, and provide a model of a phosphatase engaged with its substrate.

## Author Summary

Transplantation medicine was revolutionized by the introduction of the immunosuppressant drugs cyclosporin A and FK506 that prevent rejection of transplanted organs by the recipient's immune system. These drugs work by inhibiting calcineurin, a conserved protein phosphatase. Calcineurin regulates the immune response by dephosphorylating and activating the members of the NFAT family of transcription factors, which in turn activate genes required for the antigen-dependent stimulation of T-cells. Despite its biological and clinical importance, we have only a limited understanding of how calcineurin and other protein phosphatases interact with their substrates and target specific phosphorylated residues for dephosphorylation. Here, we determined the structure of calcineurin in complex with A238L, a viral peptide inhibitor of its function. This study shows that the viral peptide inhibits calcineurin not by targeting its active site but rather by occupying two critical substrate-binding regions of calcineurin (distant from each other and from the active site), thereby preventing its interaction with protein substrates. These findings allow us to present the first computational model of calcineurin bound to a phospho-substrate at its active site. Furthermore, by elucidating the structural basis for one particular mode of substrate–calcineurin interaction, this study reveals that both this viral peptide and immunosuppressant drugs inhibit calcineurin by blocking substrate access to a single critical region of the enzyme.

## Introduction

The vast majority of eukaryotic proteins are phosphorylated, and this modification rapidly and reversibly modulates protein dynamics, interactions, activities, localization, and/or stability [1]. This essential regulation is carried out by the opposing activities of a large array of protein kinases, and a surprisingly small cadre of phosphoprotein phosphatases. Despite decades of investigation, basic questions about how these phosphatases act on phosphosites that share little similarity in primary sequence remain unanswered [2]. Here, we unveil a key mechanism of substrate recognition by calcineurin (CN) [3], the highly conserved Ca^2+^/calmodulin-activated ser/thr phosphatase [also called Protein Phosphatase 2B (PP2B) or Protein Phosphatase 3 (PP3)]; establish that structurally unrelated inhibitors of CN specifically disrupt this interaction; and show that substrates engaged at this site have additional interactions to orient the phosphosite toward the catalytic center of the enzyme.

CN is ubiquitously expressed and is particularly abundant in the brain. By dephosphorylating a variety of protein substrates in response to Ca^2+^ signals, CN regulates development, learning and memory, cardiac function, and the immune response [3]. One of the best-studied activities of CN is its dephosphorylation of the nuclear factor of activated T-cell family of transcription factors (NFATc1-c4), which allows NFAT to translocate to the nucleus where it induces the expression of genes required for T-cell activation [4]. Because inhibition of NFAT signaling suppresses T-cell activation, natural products that specifically inhibit CN, cyclosporin A (CSA) and FK506, are widely prescribed as immunosuppressants to prevent posttransplant organ rejection [5].

CN is a member of the PPP family of protein phosphatases, which also includes PP1 and PP2A, among others. These enzymes contain structurally related catalytic domains, which rely on coordinated metal ions to directly bind phosphate and hydrolyze phosphoserine/phosphothreonine [2]. Despite their similarities, substrate recognition by these phosphatases is distinct, and natural products selectively inhibit each enzyme. In contrast to PP1 and PP2A, whose catalytic subunits combine with different regulatory subunits to create a suite of distinct holoenzymes [2], CN is always composed of a catalytic A subunit (CNA), bound to a regulatory B subunit (CNB) that binds four Ca^2+^ ions [6]. The C-terminus of CNA also contains a calmodulin-binding domain and an auto-inhibitory domain (AID), which regulate CN activity. Under basal Ca^2+^ conditions, the AID interacts with the catalytic center and prevents dephosphorylation. During signaling, increased Ca^2+^ levels cause Ca^2+^-loaded calmodulin to bind CNA, which displaces the AID from the catalytic site and stimulates CN phosphatase activity [7].

Despite the critical biological importance of CN, the molecular mechanisms that allow CN to recognize and dephosphorylate specific protein substrates are still not well understood. Some but not all substrates contain a short CN-binding motif, termed “PxIxIT,” for its consensus sequence in NFATc1-c4 [8]. This sequence, which also occurs in scaffold proteins—that is, AKAP79 [9]—binds to CNA at a groove distal from the catalytic center. PxIxIT peptides bind with equal affinities to the inactive or active form of CN [10]. Because they do not occlude or alter the CN active site, they fail to inhibit dephosphorylation of either a model phosphopeptide substrate or a small molecule, p-nitrophenyl phosphate (pNpp) [11],[12]. Thus, while this interaction can improve dephosphorylation efficiency by tethering a substrate to CN, it is not essential to the mechanism of dephosphorylation.

There has been limited insight into which substrate features do influence catalysis. Short peptides are not efficiently acted upon by CN; however, phosphopeptides that contain a basic residue at the −3 position relative to the phosphosite show 4-fold better dephosphorylation [13]. Examination of one protein substrate, the RII regulatory subunit of PKA, defined a 19 mer as the smallest peptide that was robustly dephosphorylated (K_m_ = 26 µM; V_max_ = 1.7 µmol min^−1^ mg^−1^), and showed that N-terminal residues (DLDV) lying 10 amino acids upstream of the phosphosite, were critical for substrate recognition [14]. Subsequent studies of the NFAT family (NFATc1–c4) similarly identified a conserved CN-interaction site in these proteins, “ΦLxVP,” which interacts with Ca^2+^/calmodulin-activated CN, and contributes to efficient dephosphorylation [8]. However, this sequence is significantly displaced from and C-terminal to CN-regulated phosphosites. Thus, the molecular details of the “ΦLxVP”–CN interaction and its role in dephosphorylation are still unclear.

Finally, CN is inhibited by the fungal-derived immunosuppressant drugs CSA and FK506, which bind the immunophilin proteins cyclophilin and FKBP, respectively, and engage CN as drug-immunophilin complexes. Structural analyses revealed that, unlike the AID, these drug-immunophilin complexes do not target the active site, but instead bind in a pocket ∼30 Å away at the interface of the CNA/CNB subunits [6],[15],[16]. However, while pNpp can be readily dephosphorylated by CN:drug-immunophilin complexes, CN phosphoprotein and phosphopeptide substrates cannot [17], suggesting that the presence of the drug-immunophilin complexes impedes substrate/CN binding [12]. The detailed mechanism by which immunosuppressants achieve CN inhibition is still unclear [18],[19].

Here, we describe the first high-resolution structure of CN bound to a physiological binding partner: the protein inhibitor A238L from African swine fever virus (ASFV), a highly virulent double-stranded DNA virus that infects domestic pigs in Africa and Europe. Upon infection, A238L suppresses the host immune response by inhibiting both NFkB and CN. While A238L contains a “PxIxIT”-type anchoring sequence, the molecular mechanism by which A238L inhibits the enzyme is unknown [20]. Our studies demonstrate that A238L competitively inhibits CN and that residues in A238L (“FLCV”) directly compete with substrates for binding to a substrate-recognition cleft, the ΦLxVP cleft, in activated CN. Our 1.7 Å crystal structure of the CN-A238L complex reveals the molecular interactions that mediate this key element of substrate recognition and surprisingly shows that A238L effectively inhibits CN not by blocking and occluding the active site, which is fully accessible in the complex, but instead by binding and blocking the ΦLxVP substrate recognition groove. This structure enabled molecular dynamics (MD) modeling of a minimal substrate, the RII peptide, bound to CN. The model reveals that interactions at the hydrophobic substrate-recognition groove are augmented by charged interactions at position −3 upstream of the phosphosite [13]. These studies provide the first structural insights into active site substrate engagement for any ser/thr phosphatase. Thus, this work elucidates a key mechanism by which CN recognizes substrates and provides structural insights into the presentation of phosphosites to the active site during dephosphorylation. Furthermore, the CN-A238L structure also unequivocally shows that the LxVP sequence and immunosuppressants bind to the identical site on CN. Thus, these studies establish the mechanism of action of these drugs and lay the foundation for renewed efforts in the structure-based targeted design of novel CN inhibitors.

## Results

### A238L Engages CN Via Both an LxVP and a PxIxIT Docking Motif

**Figure 1:**
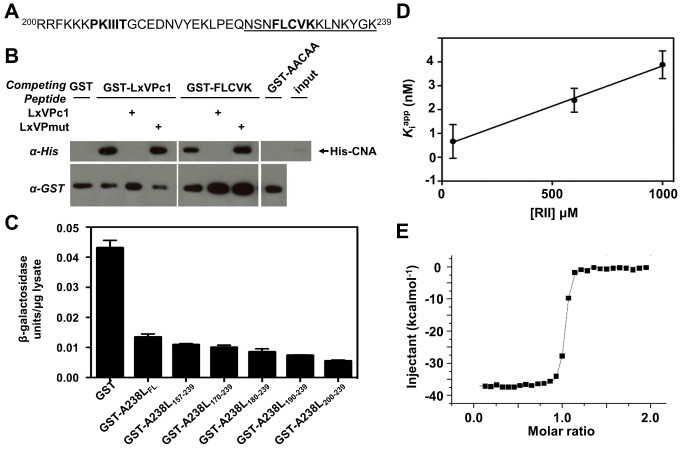
A238L interacts with CN via an LxVP and a PxIxIT motif. (A) C-terminal residues (200–239) of A238L showing putative docking site FLCVK (aa 228–232). Underlined residues were fused to GST. (B) Recombinant CN was incubated with GST fused to 15 amino acids encoding the LxVP motif of NFATc1 or the FLCVK sequence in A238L. CN co-purifies with both motifs; this interaction is disrupted by incubation with excess peptide LxVPc1 encoding the LxVP motif from NFATc1, but not LxVPmut. CN fails to co-purify with GST fused to mutated FLCVK sequence (FLCVK mutated to AACAA). (C) β-galactosidase activity of extracts from yeast strains that harbor 2xCDRE-lacZ, a CN-dependent reporter gene, and GST or GST-A238L truncations are shown. We added 50 mM CaCl_2_ to the cell culture 2.5 h before harvesting to induce CN-dependent activation of the Crz1 transcription factor ([22]; see also Text S1). Error bars indicate ± s.d. from three independent experiments. (D) Secondary plot of K*_i_* ^app^ as a function of [RII] for A238L_200–239_ inhibition of CN. Data show a linear dependence characteristic of competitive inhibition, with K*_i_* = 0.37 nM. K*_i_* ^app^ values were obtained from the nonlinear fit of Figure S1B. Points represent averages ± s.e.m. (E) Isothermal titration calorimetry confirming that purified A238L_200–239_ binds to CN.

To understand how substrates engage CN, we investigated A238L (aa 157–239, Malawi LIL20-1 strain), a protein inhibitor from the African swine fever virus (ASFV) that suppresses the host immune response by inhibiting both NFkB and CN [20]. Two substrate motifs, PxIxIT and LxVP, have been shown to bind to CN [8]. While it was known that a C-terminal fragment of A238L contains a PxIxIT motif that anchors the protein to CN [21], we showed that excess amounts of a peptide that encodes the LxVP docking motif from NFATc1, but not a mutant (LxVPc1mut LAVP→AAAA) also disrupted A238L-CN binding (Figure S1A). This establishes that A238L also contains an LxVP motif. The only sequence in A238L_157–239_ that is similar to the LxVP motif is LCVK (Figure 1A). To verify that this sequence binds CN, we generated a GST fusion protein containing the LCVK sequence from A238L (but lacking the PKIIIT sequence) and showed that it forms a complex with recombinant CN that is disrupted by the addition of a peptide encoding the LxVP motif from NFATc1, but not a mutant version of this peptide (Figure 1B). In contrast, a GST-fusion protein with a mutated sequence (FLCVK→AACAA) did not bind CN (Figure 1B). Next, we expressed full-length and truncated forms of A238L in yeast as GST fusions and measured their ability to inhibit expression of a CN-dependent reporter gene, CDRE-lacZ [22]. This analysis showed that a shorter A238L fragment, A238L_200–239_, which includes both CN binding motifs (^206^PKIIIT^211^ and ^229^LCVK^232^), is sufficient to inhibit CN (Figure 1C). Moreover, this same fragment competitively inhibits dephosphorylation of an RII phosphopeptide substrate by CN with a K_i_ of 0.37 nM (Figures 1D and S1B; RII contains an LxVP motif) [14]. A238L_200–239_ also forms a very tight complex with CN, with a dissociation constant (K_D_) of 4 nM as determined using isothermal titration calorimetry (ITC) measurements (Figures 1E and S2, and Table 1). In agreement with this result, the CN-A238L complex is a stable trimer (CN_A1–370_,_B1–170_-A238L_200–239_; hereafter referred to as CN-A238L), as evidenced by the elution of the complex in a single peak during size exclusion chromatography at the expected elution volume for a 67.2 kDa trimeric complex. Taken together, these observations demonstrate that the competitive protein inhibitor A238L_200–239_, hereafter referred to as A238L, binds tightly to CN using both a PxIxIT-type anchoring sequence and an LxVP motif.

**Table 1:** Thermodynamic parameters and dissociation (K_D_) and inhibition (K_i_) constants for CN_A1–391/B1–170_ with A238L_200–239_ wild-type, and A238L PxIxIT and LxVP mutants, derived from ITC experiments at 25°C or enzyme assays performed with RII at 37°C.

Complex	K_D_ (nM)	ΔH (kcal·mol^−1^)	−TΔS (kcal·mol^−1^)	ΔG (kcal·mol^−1^)	K_i_ (nM)
CN:A238L_WT_	4±1	−40.3±4.9	28.7±4.9	−11.5±0.2	0.37±0.03
CN:A238L_PKIIITmut._	624±26	−34.3±0.9	25.9±0.9	−8.5±0.0	15±1
CN:A238L_FLCVKmut._	803±26	−14.0±0.1	5.7±0.1	−8.3±0.0	7,700±3,000

Thermodynamic and dissociation constant data represent mean values ± one s.d. for triplicate measurements except A238L_WT_, which was performed 5 times. Inhibition constants are mean values ± one s.e.m. from three independent experiments.

### Structure of the CN-A238L Heterodimer/Inhibitor Complex

**Figure 2:**
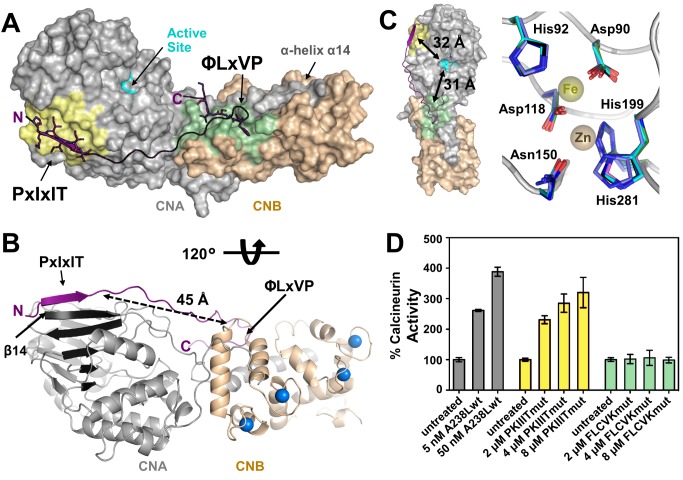
The CN active site is fully accessible in the CN-A238L complex. (A) Overview of the CN-A238L complex. CNA (gray, surface representation) interacts via helix α14 with CNB (beige, surface representation). The CNA active site is highlighted in cyan, with PxIxIT and LxVP binding pockets shown in yellow and green, respectively. A238L is shown as a cartoon representation (purple), with the PxIxIT (PKIIIT) and LxVP (LCVK) substrate binding motifs highlighted as sticks. (B) Cartoon representation of the CN-A238L complex. Structure is rotated 120° about the *x*-axis relative to (A); all colors as in (A); blue spheres representing four Ca^2+^ atoms in CNB. CNA β-sheet1 is shown in dark grey. The N-terminal β-strand formed by the A238L PxIxIT motif complements β-strand14 of CNA and thus extends β-sheet1. (C, left) CN-A238L illustrated as in (A), with the distances between the CN active site and the PxIxIT and LxVP binding grooves indicated by arrows (∼32 Å and ∼31 Å, respectively). (C, right) Overlap of the catalytic residues from the CN-A238L complex (black), apo-CN (cyan, PDBID 1AUI), CN-AKAPpeptide (dark blue, PDBID 3LL8), CN-PVIVITpeptide (green, PDBID 2P6B), CN-Cyclosporin (light blue, PDBID 1M63), and CN-FK506 (pink, PDBID 1TCO). Catalytic residues of CNA are shown as sticks and labeled (Asp90, His92, Asp118, Asn150, His199, His281). Fe^3+^ and Zn^2+^ ions from 1AUI are shown as spheres. (D) The rate of pNpp hydrolysis was measured in the presence of A238L (grey), A238L_PKIIITmut_ (yellow), or A238L_FLCVKmut_ (green). Assays were performed in triplicate using 10 mM pNpp, and error bars indicate one s.d.

To elucidate the molecular mechanism of A238L binding and inhibition, we determined the 1.7 Å crystal structure of the CN-A238L heterodimer/inhibitor complex (CNA/CNB/A238L). The structure of the CNA/B heterodimer in complex with A238L is virtually identical to CNA/B heterodimer structures from previous reports [6],[9],[15],[16],[23]–[25]. CNA residues 1–13, CNB residues 1–5/161–170, and A238L residues 200–204/235–239 were not visible in the electron density map and thus were not modeled. The absence of electron density for A238L residues 200–204 (N-terminal to the PxIxIT motif) and A238L residues 235–239 (C-terminal to the LxVP motif) suggests that these regions remain flexible upon complex formation and do not contribute to CN binding. A238L binds CN in a largely extended conformation, stretching from the PxIxIT binding site to the CNA/CNB interface and then looping back along the CNA/B interface to occupy the newly identified LxVP binding pocket (Figure 2A, B). As a consequence, the CN-A238L interaction buries 3,083 Å^2^ of solvent accessible surface area (SASA). Because A238L potently inhibits CN, it was predicted that A238L would bind and occlude the CN active site. However, the structure reveals that the CN active site is fully accessible in the CN-A238L complex (Figure 2C, left). Moreover, the key catalytic residues are structurally invariant when compared with those in previously determined CN structures [6],[16],[24], suggesting that CN in the CN-A238L complex is catalytically active (Figure 2C, right). The ability of CN to dephosphorylate small molecule substrates was confirmed using p-nitrophenyl phosphate (pNpp) dephosphorylation assays (Figure 2D). In fact, A238L and A238L_PKIIITmut_ both increased the rate of pNpp hydrolysis compared to untreated CN, consistent with previously reported rate increases by both a peptide containing the LxVP sequence from NFATc1 as well as by the immunosuppressants FK506 and CSA [12],[17]. Thus, the CN-A238L complex retains full catalytic activity. Together, these data show that A238L does not inhibit CN by blocking the catalytic center, but instead utilizes an alternative mechanism.

### The CN-A238L PxIxIT Interaction

**Figure 3:**
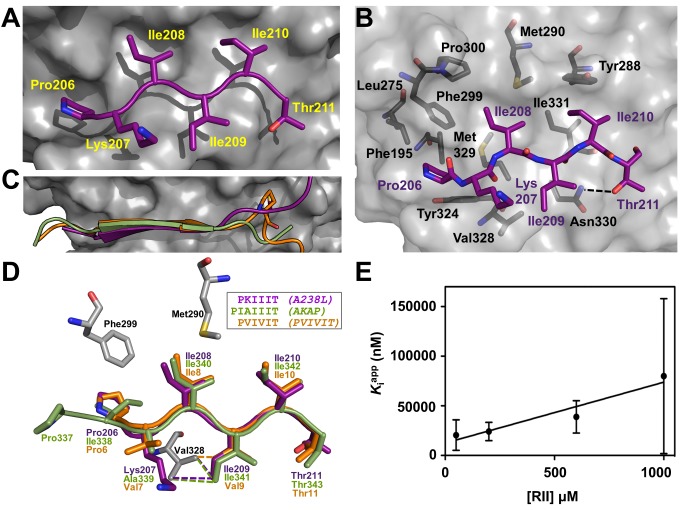
CN-A238L interactions: The PxIxIT substrate binding site. (A) Close-up view of the CN PxIxIT substrate binding site. A238L PKIIIT is shown as magenta sticks and labeled; CNA is shown as a grey surface. (B) Same view as (A), with a transparent CNA surface. Individual CNA residues that participate in the interaction with PKIIIT_A238L_ are shown as grey sticks. (C) Superposition of the PKIIIT_A238L_ motif (purple) with a synthetic PVIVIT peptide (orange) and the IAIIIT CN docking site of AKAP79 (green) bound to CN. Pro13_PVIVIT_ and Cys213_A238L_ are shown as sticks. (D) Overlay as in (C) but illustrated as sticks. Corresponding “variable” residues Ile209_A238L_, Val9_PVIVIT_, and Ile341_AKAP79_ (the second “x” in PxIxIT) participate in the same hydrophobic interaction with Val328_CNA_ (dotted lines). (E) Secondary plot of K_i_^app^ as a function of [RII] for inhibition of CN by A238L_FLCVKmut_, which retains the PKIIIT site. Data show a linear dependence characteristic of competitive inhibition, with K_i_ = 7,700±3,000 nM. K_i_^app^ values were obtained from the nonlinear fit of Figure S4A. Points represent averages ± s.e.m.

The interaction of A238L with CNA at the PxIxIT binding pocket buries 886 Å^2^ of SASA and is mediated by A238L residues 206–211 (PKIIIT, the PxIxIT motif from A238L), which form a short β-strand that hydrogen bonds with β14 of CNA to extend one of its central β-sheets (Figure 3A–C). As observed for other CN-PxIxIT_peptide_ complexes, hydrophobic contacts are the dominant determinants of specificity in the PKIIIT interaction between A238L and CNA (Figure 3B). The PxIxIT interaction in the CN-A238L complex is nearly identical to those observed in the CN-PVIVIT_peptide_ and the CN-AKAP_peptide_ complexes [9],[24], demonstrating that PxIxIT sequences, contained in many CN-interacting proteins, likely bind CN in a similar manner (Figure 3C,D). The CN-A238L complex also allows for the comparison of interactions outside the PxIxIT binding groove (Figure 3C). As expected, these interactions are more variable. For example, while the C-terminal residues of both peptides angle down away from CNB, the A238L chain continues upwards toward the CNA/B interface. Furthermore, both Pro13_PVIVIT_ and Cys213_A238L_, but not Thr345_AKAP_, bind in a shallow hydrophobic pocket comprised of the methyl groups of Lys318_CNA_, Gln333_CNA_, and Asn335_CNA_, suggesting that this pocket might be engaged by other endogenous substrates to enhance binding (Figure 3C). These results illustrate how sequence variations not only in the PxIxIT sequence itself but also in flanking residues may fine-tune the affinity of PxIxIT sequences for CN [26].

### The CN-A238L LxVP Interaction

**Figure 4:**
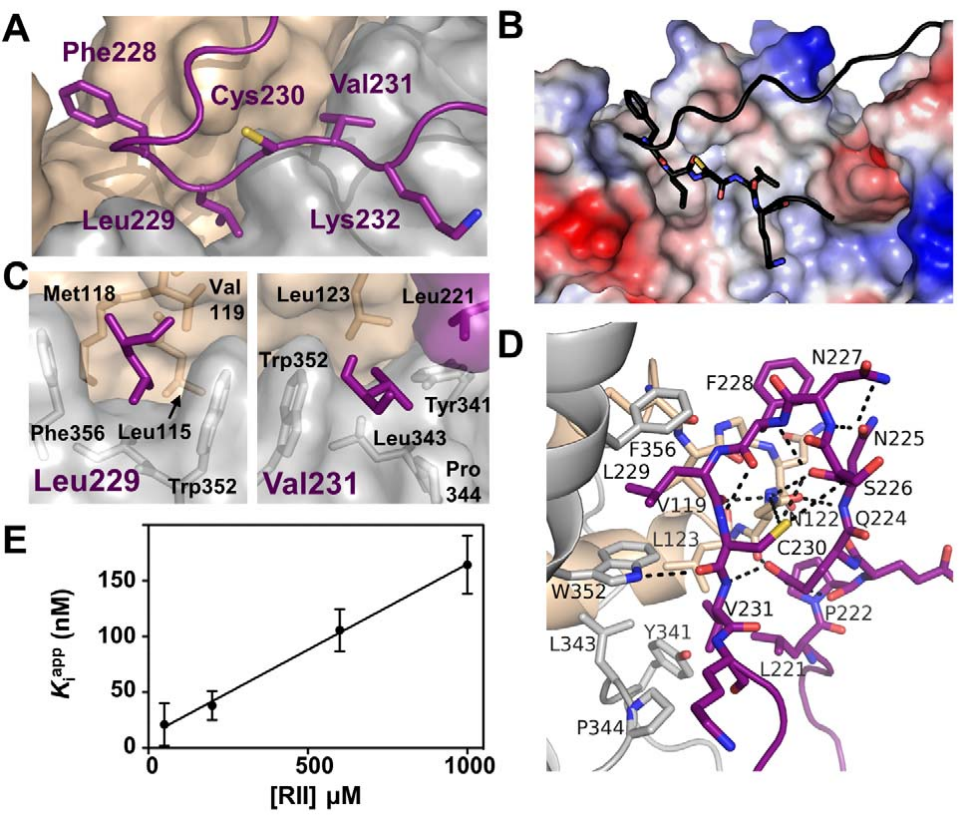
CN-A238L interactions: The LxVP substrate binding site. (A) Close-up view of the CN ΦLxVP substrate binding site. (B) Electrostatic surface potential of the A238L-CN complex, highlighting the hydrophobic nature of the ΦLxVP binding groove. (C) CN residues that make up the Leu229 hydrophobic binding pocket (left) and the Val231 hydrophobic binding pocket (right). (D) Electrostatic intermolecular interactions between CN and A238L and intramolecular interactions at the A238L kink, which coordinate A238L for ΦLxVP binding. (E) Secondary plot of *K*_i_^app^ as a function of [RII] for inhibition of CN by A238L_PKIIITmut_, which retains the FLCVK site. Data show a linear dependence characteristic of competitive inhibition, with *K*_i_ = 15 nM. Points represent averages ± s.e.m. from three independent experiments.

A short motif, LxVP, mediates interaction of several substrates, as well as A238L, with Ca^2+^/calmodulin-activated CN [12]. However, until now, the molecular interactions that mediate LxVP recognition by CN have remained unknown (Figure 4). In the CN-A238L complex, the LxVP motif from A238L, ^229^LCVK^232^, is bound to CN (Figure 4A), revealing the very hydrophobic LxVP binding pocket at the CNA:CNB interface in CN (Figure 4B). When bound to CN, the A238L LCVK residues are extended, burying 728 Å^2^ of SASA. Unlike the CN PxIxIT binding pocket, which is comprised of residues only from CNA, the CN LxVP binding pocket contains residues from both CNA and CNB. Leu229_A238L_, which becomes 92% buried upon complex formation, makes hydrophobic contacts with residues from both CNA and CNB subunits: Trp352_CNA_ and Phe356_CNA_, which, when mutated, alter binding to the LxVP sequence from NFATc1 [12], as well as Leu115_CNB_, Met118_CNB_, and Val119_CNB_ (Figure 4C, left). Similarly, Val231_A238L_ becomes 97% buried when bound to CN, forming hydrophobic interactions with Tyr341_CNA_, Leu343_CNA_, Pro344_CNA_, Trp352_CNA_, and Leu123_CNB_ (Figure 4C, right). In addition, Cys230_A238L_ (the “x” in LxVP) forms hydrogen bonds with Trp352_CNA_ and Asn122_CNB_ (Figures 4D and S3A). Finally, Lys232_A238L_, which is a noncanonical residue in the LxVP motif as it has a “K” instead of the expected “P,” does not interact with CN and thus is not important for CN recognition by A238L. Consequently, this work shows that residues Leu229_A238L_ and Val231_A238L_, which make significant interactions with both CNA and CNB, are the key residues that mediate binding to the LxVP interaction pocket. Notably, Phe228_A238L_, which is immediately N-terminal to the LxVP binding motif, also contributes to CN binding (Figure 4A, B), fitting into a deep pocket formed by the loops connecting EF-hands 1 and 2 and EF-hands 3 and 4 of CNB. This results in a 25% increase in the SASA buried at this site (FLCVK buries 911 Å SASA). Critically, multiple LxVP sites are immediately preceded by an aromatic residue (Phe or Tyr), which may act as a binding strength enhancer, as mutation of this aromatic residue weakens the LxVP interaction [12]. Thus, a subset of LxVP sites, including the one in A238L, is best described as ΦLxVP.

### A238L Residues Also Contribute to the LxVP Binding Pocket

The ΦLxVP binding groove is comprised of residues from both CNA and CNB, in the CN-A238L complex. Unexpectedly, however, a few A238L residues also contribute to the ΦLxVP binding pocket. Specifically, A238L, which projects upwards from the PxIxIT site towards the CNB interface, forms a tight 180° turn at Asn227_A238L_ (Figures 4D and S3A). This kink is essential for ΦLxVP binding, as it redirects the ΦLxVP sequence (FLCVK) back toward its docking site on CN at the CNA/B interface. This enables the A238L residues that immediately precede the ΦLxVP to interact directly with the CN-bound ΦLxVP residues and contribute to the ΦLxVP binding site. Thus, in addition to the multiple interactions observed with residues from CNA and CNB, Val231_A238L_ (the “V” in the LxVP motif) also makes intramolecular hydrophobic contacts with Leu221_A238L_. In addition, the side chain Gln224_A238L_ hydrogen bonds with the backbone amide of Val231_A238L_ (Figures 4D and S3A). Therefore, residues from all three proteins—CNA, CNB, and A238L—function to keep Val231_A238L_ occluded from solvent in the bound conformation. In addition, although Cys230_A238L_ is the “x” in the LxVP motif, this residue also makes multiple intramolecular interactions that help stabilize the A238L bound conformation. Specifically, the amide nitrogen of Cys230_A238L_ hydrogen bonds with the carbonyl oxygen of Phe228_A238L_. Cys230_A238L_ also forms thiol hydrogen bonds with the amide nitrogen of S226_A238L_ and the carbonyl of N225_A238L_. These intramolecular interactions explain why Cys230_A238L_ is still nearly completely buried (70%) in the CN-A238L complex even though its side chain points away from the LxVP docking groove. Thus, although it is not yet known how similar the CN-LxVP interaction of CN-A238L is with that of other LxVP docking motifs from substrates, our structure suggests that residues flanking the LxVP sequence may also modulate the affinity of this motif for CN.

Although the most extensive interactions between A238L and CN occur at the PxIxIT and ΦLxVP binding grooves, additional, largely polar intra- and intermolecular interactions outside of these docking sites also contribute to A238L binding. For example, the interactions that stabilize the A238L kink (the 180° tight turn at Asn227_A238L_, which enables the rest of A238L to point back toward the LxVP binding pocket) are mediated by an extensive network of more than 10 hydrogen bonds, the center of which is Asn122_CNB_ (Figures 4D and 3SA). The side chain amide nitrogen and carbonyl of Asn122_CNB_ form hydrogen bonds with the backbone carbonyl and backbone amide nitrogen of two residues that border the kink, L229_A238L_ and Asn225_A238L_, respectively. Similarly, A238L residues C-terminal to the PxIxIT motif (^212^GCEDNVY^218^) also interact with CNA through main chain/side chain side chain/side chain hydrogen bonds (Figure S3B). Although the two dominant A238L:CN PxIxIT and LxVP interfaces together bury 1,797 Å^2^ SASA, these additional mostly polar interactions also contribute to the CN-A238L interface, burying 1,286 Å^2^ SASA.

### A238L Inhibits CN by Interfering with Substrate Recognition

**Figure 5:**
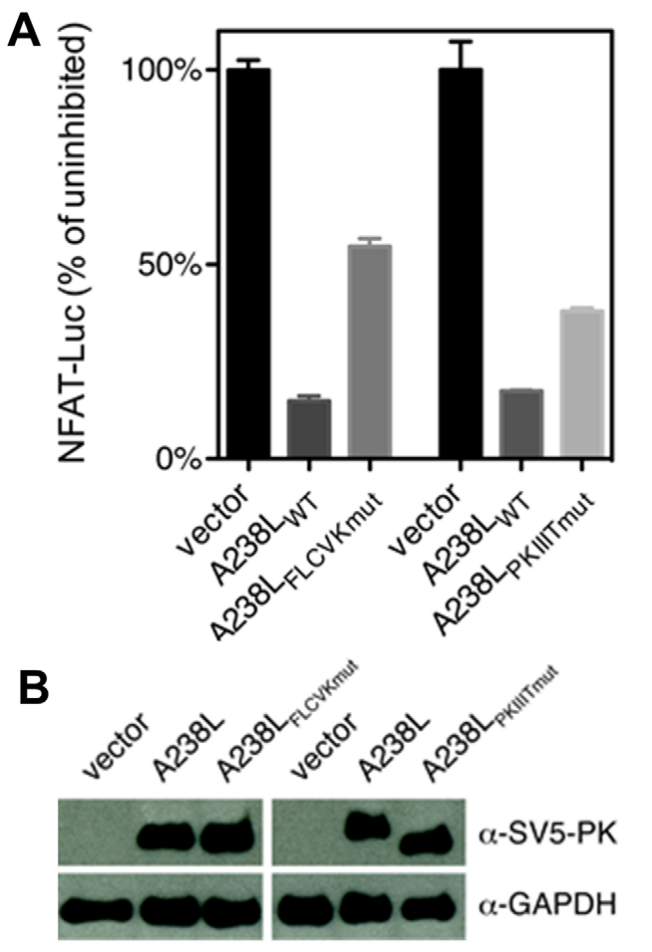
A238L inhibits CN by interfering with substrate docking. (A) NFAT-dependent transcription was measured in stimulated HEK293T cells co-transfected with an NFAT-luciferase reporter plasmid and a plasmid expressing SV5-PK-tagged A238L wild-type or motif mutant proteins, or vector alone. Data are means ± s.e.m. from three independent experiments. (B) Transiently transfected SV5-PK-tagged A238L protein expression levels were analyzed by immunoblotting. The increased electrophoretic mobility of A238L_PKIIITmut_ relative to wt-A238L may be due to changes in a posttranslational modification that is known to affect the electrophoretic mobility of A238L [55].

We next sought to determine the relative importance of the PxIxIT and LxVP sites for CN binding. Using ITC and two A238L mutants (a PxIxIT motif mutant, with PKIIIT mutated to AKAIAA and a ΦLxVP motif mutant, with FLCVK mutated to AACAA; A238L_PKIIITmut_ and A238L_FLCVKmut_, respectively), we found that both the PxIxIT and ΦLxVP sequences are important for CN binding, as the PxIxIT and ΦLxVP mutations increase the K_D_ similarly by ∼150-fold and ∼200-fold, respectively (Table 1 and Figure S2). Next, we investigated the contribution of the individual A238L motifs toward the inhibition of CN. A238L_PKIIITmut_, which still contains the FLCVK sequence, competitively inhibits dephosphorylation of RII by CN with K_i_ = 15 nM (Table 1 and Figure 4E) [14]. In contrast, A238L_FLCVKmut_, which still contains the PKIIIT sequence, inhibited CN very poorly, with K_i_ = 7,700 nM, a 20,000-fold decrease in inhibitor efficacy compared to wild-type A238L (Table 1 and Figures 3E and S4). In addition, when expressed in HEK293T cells in which NFAT signaling had been stimulated, both A238L mutants reduced the activity of an NFAT-dependent reporter gene. At roughly equal levels of protein expression, neither A238L_FLCVKmut_ nor A238L_PKIIITmut_ were as effective as wt-A238L in NFAT inhibition (Figure 5A, B). Taken together, these results show that in A238L, FLCVK, but not PKIIIT, is required to competitively inhibit dephosphorylation of the RII phosphopeptide, whereas both sequences contribute to reducing CN-mediated dephosphorylation of substrates, such as NFATs, that contain an LxVP substrate recognition motif, and also require PxIxIT-mediated anchoring to CN. Therefore, A238L does not directly inhibit CN activity by blocking its active site, but instead inhibits CN via a model of steric occlusion where it blocks the access of substrates to key binding sites on CN, similar to the steric occlusion of substrate selection previously reported for Protein Phosphatase 1 [27].

### Model of CN-Substrate Dephosphorylation

**Figure 6:**
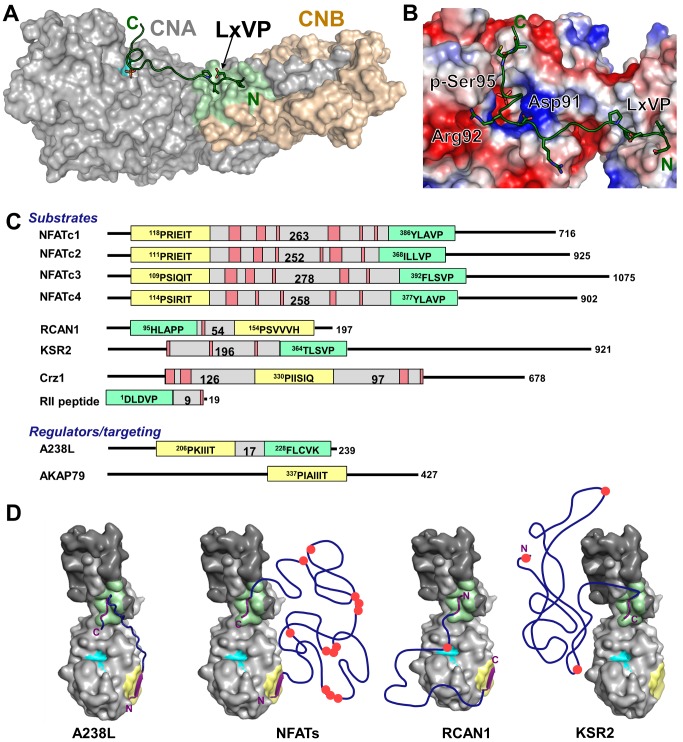
Potential interaction modes of CN substrates/regulators with CN. (A) The CN-RII peptide complex obtained by MD. Colors as in Figure 2A, C. CN is shown in surface representation and the RII peptide in dark green with the LxVP motif (LDVP) and phospho-Ser95 as green sticks. LDVP is bound to the LxVP binding pocket (light green), and phospho-Ser95 is bound in the CN active site (cyan). (B) Electrostatic interactions between CN and the RII peptide. The CN electrostatic surface has positively and negatively charged areas colored blue and red, respectively. The LxVP motif and residues in RII that participate in polar interactions with CN are shown as green sticks. (C) Features of selected CN substrates and regulators, including substrates tested in this work (NFAT, Crz1, and the RII peptide). PxIxIT and LxVP motifs are highlighted in yellow and green, respectively, with intervening residues in grey. Regions containing S-T residues that are dephosphorylated by CN are pink. (D) Potential modes of interaction of CN with various binding partners. CN is shown in grey, with the active site in cyan, the PxIxIT docking site in yellow, and the LxVP docking site in green. CN binding partners are shown in blue, with PxIxIT and LxVP motifs in purple and phosphorylated regions shown as red circles. The residues between the two CN docking motifs, or between one docking motif and regions dephosphorylated by CN, are represented as coils, as they are predicted to be unstructured in solution. A238L is the CN-A238L crystal structure.

The CN-A238L structure revealed the molecular interactions that mediate CN recognition of the LxVP motif, and we showed that the RII phosphopeptide requires this interaction for its dephosphorylation; therefore, we used bias-exchange metadynamics MD [28] to generate the first detailed model of a substrate, the RII phosphopeptide, bound to activated CN and poised for dephosphorylation (Figure 6A). The LxVP sequence of the RII peptide (^81^D**L**D**VP**IPGRFDRRV***pS***VCAE^99^) is separated from the phospho-Ser95 residue by nine amino acids, which, due to the distance between the LxVP-binding site and the catalytic center, limits the potential conformations this peptide can adopt during dephosphorylation. Our model of the CN:RII peptide complex shows that the interaction of RII (^82^LDVP^85^) with the CN LxVP site is very similar to that observed in the CN-A238L complex. It also shows that additional electrostatic and hydrophobic interactions stabilize interactions between CN and the intervening RII residues, which function to guide p-Ser95 to the CN active site. In particular, RII residues Asp91 and Arg92 form a number of key side chain hydrogen bonds and salt bridge interactions with CNA (Figure 6B). This is consistent with previous work suggesting that Arg in the −3 position is a strong positive determinant of dephosphorylation efficiency by CN [13]. The model also shows that the nine intervening residues between the LxVP and phospho-Ser95 are essential for spanning the distance between the CN LxVP binding and active sites. This demonstrates that substrates with phosphorylated residues that are close to the LxVP site (e.g., within six or fewer residues) are unlikely to be dephosphorylated by CN as they will be unable to reach the active site.

## Discussion

Our biochemical and structural studies reveal the mechanism by which A238L, a protein made by African swine fever virus that dampens the host immune response during infection, potently inhibits CN. The structure of CN (CNA_1–370_ and full-length CNB-subunit) in complex with A238L shows that 30 amino acids of A238L form a tight complex with CN. We conclude that an equivalent complex forms with full-length CN, as neither the presence of the AID nor additionally described inhibitory sequences [29] interfere with the inhibition of CN by A238L in vivo. As anticipated, A238L interacts with the PxIxIT binding groove on CNA. However, we discovered that A238L also binds CN in additional surface grooves, the most important of which is the LxVP-binding pocket. Remarkably, while A238L is a potent inhibitor of CN, the CN catalytic center is fully accessible and active, as confirmed by our biochemical findings that show that A238L-bound CN rapidly dephosphorylates the small molecule substrate pNpp. In contrast, the CN-A238L complex is unable to dephosphorylate a peptide substrate derived from the RII subunit of protein kinase A, which contains an LxVP sequence, or a protein substrate, NFAT, which contains an LxVP sequence and a PxIxIT anchoring sequence. Collectively, these structural and biochemical data show that A238L inhibits CN function through a model of “steric inhibition,” in which A238L effectively inhibits CN function, not by directly blocking the active site of the phosphatase, but instead by occupying two critical docking grooves and sterically occluding CN from interacting with substrates. To our best knowledge, this mechanism of altered substrate dephosphorylation by steric occlusion of substrate binding sites has only been directly observed in one other ser/thr protein phosphatase, that of PP1, which, like CN, is one of the key members of the PPP family [27]. Thus, our results establish that this mechanism, whereby enzyme activity is modulated via substrate access rather than through active site inhibition or allostery, is likely utilized by the entire PPP family.

Although the structure of CN bound to a bona fide substrate has yet to be determined, the CN-A238L structure and our CN-RII substrate model significantly advance our understanding of how CN uses two complementary strategies to recognize substrates. First, the PxIxIT anchoring motif is used by protein scaffolds, inhibitors, and some substrates to form a stable interaction with CN by docking to a site that is available regardless of the activation state of the enzyme. For PxIxIT-containing substrates, the strength of this anchoring modulates the Ca^2+^-concentration dependence of their dephosphorylation, but does not directly contribute to recognition of phosphosites during the dephosphorylation reaction [26],[30]. Second, CN overcomes the limited specificity of its catalytic site by recognizing specific residues, such as “ΦLxVP,” which are distal to the phosphosite. This interaction is likely required for the dephosphorylation of multiple substrates, as it is this site that is targeted by multiple inhibitors, including A238L and the immunosuppressants CSA and FK506 (Figure 6C).

This work also reveals interactions that contribute to phosphosite selection by CN. The “ΦLxVP” binding site is now molecularly defined as a hydrophobic binding surface composed of residues from the CNA and CNB subunits, which becomes accessible after binding of Ca^2+^-loaded calmodulin displaces the C-terminal AID domain from the active site [12]. Our structure and the RII model show that the substrate residue dephosphorylated by CN (hereafter referred to as pS/pT) must be a minimum of 9–15 residues away from the ΦLxVP sequence, and be in an extended conformation in order for the pS/pT residue to reach the catalytic site. In fact, the pS/pT residues in the RII peptide and the substrate RCAN1 are only 9 and 10 residues C-terminal to the “P” in their LxVP motifs, respectively (Figure 6A, B, D) [14],[31]. Our model also shows that additional electrostatic interactions, especially at basic residue at position −3, help orient the phosphosite in RII toward the catalytic center. In addition, the C-terminus of the RII peptide lies in a groove identified in other PPPs, namely PP1, to be a substrate recognition groove (hydrophobic groove in PP1), suggesting that this groove functions in a similar manner in CN and thus likely for the entire family of PPPs [2],[32].

While our model provides fundamental insights into phosphosite selection by CN for those substrates in which the phosphosite is 9–10 residues C-terminal to LxVP sequence, other surface grooves near the catalytic center are likely also important for substrate recognition. This is because the spacing between experimentally determined “LxVP” and phosphosites substrates is quite variable. For example, substrates such as NFAT and KSR2 have pS/pT sites that are 10–100 s of residues away from the known LxVP sequences [33]–[37]. In these cases, one or several pS/pT sites are found in long, extended sequences that are predicted to be unstructured, suggesting that these regions are dynamic when bound to CN and that interactions at the LxVP and PxIxIT sites tether the substrate to CN allowing the phosphosite(s) to encounter the catalytic site and be dephosphorylated (Figure 6D). A detailed understanding of how phosphosites that are distal from the LxVP and PxIxIT motifs engage the CN catalytic center is an active area of investigation.

**Figure 7:**
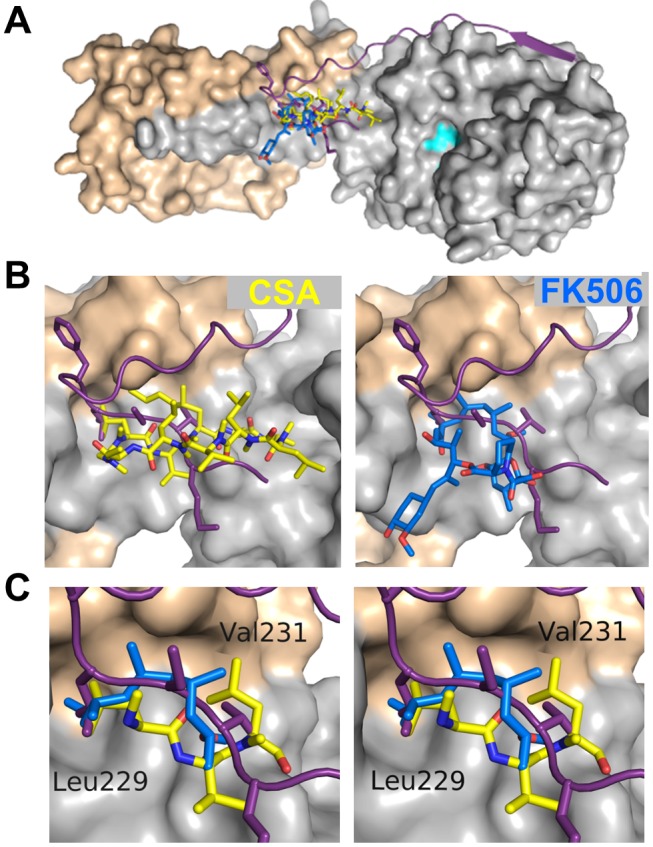
The immunosuppressive drugs FK506 and CSA inhibit CN by occupying the LxVP substrate recognition site. (A) Overlay of CN-bound A238L (purple), CSA (yellow), and FK506 (blue). (B, left) Overlay of A238L with CSA at the LxVP substrate binding groove. (B, right) Overlay of A238L with FK506. (C) Stereoview of the Leu229 and Val231 binding pockets in the LxVP substrate binding groove with A238L, CSA, and FK506. Some atoms of CSA and FK506 removed for clarity.

Finally, these studies also provide critical new insights into the mechanism by which the immunosuppressants CSA and FK506 inhibit CN. Our structure reveals that the location of the LxVP substrate-binding site on CN is identical to that of the CSA/FK506 binding site (Figure 7A, B). Furthermore, we identified several contacts common to all of these interactions. In particular, multiple residues from the cyclic immunosuppressant molecules overlap nearly perfectly with key residues of the A238L LxVP sequence, LCVK, especially at Leu229_A238L_ and Val231_A238L_, and occupy hydrophobic surfaces formed by Trp352 and Phe356 of CNA and Met118 and Val119 of CNB (Figure 7C). These findings indicate that disrupting the interaction between CN and LxVP motifs found within substrates is sufficient for inhibiting the dephosphorylation of LxVP-containing substrates, and explains the molecular mode of action of these drugs. Furthermore, the ability of FK506 and CSA to antagonize all known functions of CN suggests that every substrate contains at least one LxVP motif and that interaction of this motif with CN is essential for substrate dephosphorylation. Consequently, our high-resolution 3-dimensional structure of the CN-A238L complex opens up a new avenue for the development of a novel class of powerful CN inhibitors that selectively and potently bind the CN LxVP binding site. Both CN substrate-binding pockets, but especially the LxVP pocket, present excellent targets for the development of immunosuppressive drugs. Clearly, such drugs will also be an extremely powerful tool for investigating the many regulatory processes, including immune activation and cardiac hypertrophy, that are driven by CN.

## Materials and Methods

### Proteins

CNA (residues 1–370 or 1–391), CNB (residues 1–170), and wt- and mutant A238L (residues 200–239) were subcloned into vectors containing either an N-terminal His_6_- or GST-tags and expressed in *E. coli*. To form the CN-A238L (CNA, 1–370; CNB, 1–170; A238L, 200–239) complex used for X-ray crystallography, His_6_-tagged CN was purified over a HisTrap HP column and directly eluted into an at least 2-fold molar excess of previously purified A238L. His_6_-tag cleavage and subtraction purification were performed, and the complex was further purified by SEC (Superdex 200 26/60) in 20 mM Tris pH 8.0, 50 mM NaCl, 0.5 mM TCEP, and 1.5 mM CaCl_2_. Additional cloning, protein expression, and protein purification procedures are provided in Text S1.

### Crystallization and Structure Determination

The CN-A238L complex (∼7 mg/ml) formed thin plate crystals in 0.16 M ammonium citrate and 20% (w/v) PEG3350 at 22°C. Crystals were obtained using the sitting drop vapor diffusion method (three-well Intelliplate, Art Robbins), with 0.6 µl drops containing a 1∶2 ratio of precipitant solution to protein and 50 µl of precipitant solution in the reservoir. Crystals were cryo-protected using a 10 min soak in mother liquor supplemented with 30% glycerol and immediately flash frozen in liquid N_2_. Data were collected at the NSLS X25 beamline at Brookhaven National Laboratory. Crystals of CN-A23L formed in space group P2_1_, with unit cell dimensions *a* = 72.69 Å, *b* = 48.98 Å, *c* = 82.44 Å, and *β* = 104.4. Data were indexed, integrated, and scaled with *DENZO* and *SCALEPACK* as part of *HKL2000* [38]. The structure of CN-A238L was determined to 1.7 Å by molecular replacement using a CN heterodimer molecule (CNA/B; PDBID 1AUI [6]; the auto-inhibitory domain was omitted) as the search model. The final model of the CN-A238L complex was obtained using iterative rounds of refinement in Phenix [39] and model building in Coot [40], with TLS used in the final round of refinement. The asymmetric unit contains one copy of the CN-A238L heterodimer/inhibitor complex. The final structure refined to a final R factor of 15.8% (R_free_ = 17.8%). CN-A238L crystals formed at pH 5, leading to protonation of active site residues and thus displacement of the positively charged active site metal ions, as previously seen by Jin & Harrison, who crystallized CN at pH 4.6 [16]. One hundred percent of all residues are in the allowed region of the Ramachandran diagram. Structure validation and stereochemistry analysis was performed with Molprobity [41]. Details and statistics of data analysis and model building are provided in Table S1 and Text S1. A stereoview of the A238L electron density is in Figure S5.

### MD Simulations

The CN-A238L structure was used as the starting structure for the MD simulations. The phosphoserine (pSer) was placed in the active site according to the PDB structure 1TCO, together with the expected OH ligand [23]. We used density functional theory (DFT) with the B3LYP functional [42], as implemented in TURBOMOLE [43], to obtain an energy minimized structure of the CN active site, including the partial charges of the active site metal ions and their ligands. During the MD simulations, the active site region was restrained to the DFT-optimized configuration. Initial structures of RII bound to CN were built according to the backbone coordinates of A238L at the LxVP site, and the last eight residues of the auto-inhibitory domain in PDB structure 1AUI [6]. Ten different starting models for the intervening sequence (^86^IPGRFD^91^) were constructed using the ROSETTA loop model tool [44]. The resulting 10 CN-RII complexes were inserted into rectangular boxes (8×8×12 nm^3^) with 22,881 TIP3P water molecules [45] and eight Na^+^ ions each for electro-neutrality. MD simulations were performed using GROMACSv4.5.3 [46], the Amber ff99SB-ILDN force field [47], particle mesh Ewald summation [48] using a 0.12 nm grid spacing, a time step of 2 fs and a 0.9 nm real-space cutoff at a constant temperature [49] of 300 K and at a pressure [50],[51] of one bar. After 2 ns of equilibration of each of the 10 starting models, bias-exchange metadynamics [28] using the PLUMED1.3.0 plugin [52] was used to accelerate the conformational sampling. In 9 of the 10 replicas, the Ψ dihedral angles of RII residues Ile86, Pro87, Arg89, Phe90, Asp91, Arg92, Arg93, Cys97, and Ala98, respectively, were biased, while replica 10 was kept unbiased. To ensure RII stayed bound to CN during the conformational sampling, the Fe^3+^-pSer and LxVP interactions were harmonically restrained. Convergence was reached after 1.15 µs (115 ns/replica), as judged by the free energy profiles of the biased Ψ. The RII structures of the unbiased replica were clustered according to a 1.2 Å backbone root-mean-square-distance (RMSD) threshold. The most populated cluster contains 58% of all structures, from which a representative was selected with the lowest Coulomb energy. A different clustering technique using reweighting of the biased replicas [53] led to similar results.

### Isothermal Titration Calorimetry

ITC experiments were performed at 25°C using a VP-ITC microcalorimeter (GE Healthcare). All protein samples were equilibrated in ITC buffer (20 mM Tris pH 7.5, 150 mM NaCl, 1.5 mM CaCl_2_, 0.5 mM TCEP). Wild-type or mutated A238L was titrated into CN_A1–391/B1–170_. Titrant (10 µL per injection) was injected into the sample cell over a period of 20 s with a 250 s interval between titrations to allow for complete equilibration and baseline recovery. Twenty-eight injections were delivered during each experiment, and the solution in the sample cell was stirred at 307 rpm to ensure rapid mixing. Data were analyzed with one set of sites binding model, based on the 1∶1 stoichiometry observed in the crystal structure, using Origin 7.0 (OriginLab).

### CN Activity Assays with RII

The rate of RII phosphopeptide dephosphorylation by CN was determined by measuring the total phosphate released over four time points (total 5–20 min). Reaction rates were linear over this time period and constituted less than 1% of product formation. The 50 µl reactions contained assay buffer (50 mM Tris pH 7.4, 100 mM NaCl, 6 mM MgCl_2_, 0.5 mM CaCl_2_, 0.1% PEG 3250, 0.5 mM DTT), 10 nM CN, wt- or mutant A238L (0–10 µM), and 50–1,000 µM RII phosphopeptide. Reactions were performed at 37°C and were initiated by the addition of 10× RII and terminated using 100 µl Biomol Green Reagent (Enzo Life Sciences). After color development for 20 min, absorbance was measured at 595 nm and compared to phosphate standards of known concentration to determine the amount of phosphate released. Kinetic constants were determined with GraphPad Prism by fitting the points to the Morrison model for tight-binding inhibitors using nonlinear regression analysis [23]. Competitive and noncompetitive models were compared using the corrected Akaike information criterion, AICc [54]. Phosphorylated RII was synthesized by the Tufts University Core Facility.

### CN Activity Assays with pNpp

The rate of CN hydrolysis of pNpp was measured in a continuous assay by monitoring the production of pNP at 415 nm. Reaction rates were linear for at least 1 h and reaction progress was <0.1%. The 100 µl reactions contained assay buffer (100 mM Tris pH 7.5, 100 mM NaCl, 0.4 mM CaCl_2_, 100 µg/ml BSA, 1 mM MnCl_2_, 0.5 mM DTT), 10–20 nM truncated CN, wt or mutant A238L (0–8 µM), and 10 mM pNpp. Experiments were performed at room temperature. Standards of known pNp concentration were used to convert absorbance units to pNp concentration.

### GST Pull-Down and Competition Assays

Cells extracts were prepared by resuspending cell pellets in lysis buffer (50 mM Tris, pH 7.4, 100 mM NaCl, 2 mM EDTA, 2 mM EGTA, 5 mM DTT, 1 mM PMSF, 5 µg/ml each pepstatin, leupeptin, aprotinin, and benzamidine) and lysed by sonication. Cell debris was pelleted by centrifugation (20,000× *g*, 20 min), and clarified lysate was brought to 0.1% Tween-20 and stored in aliquots at −80°C. For GST-peptide fusion experiments, 50–200 µg cell extracts containing GST or GST fusion proteins were bound to glutathione sepharose 4B beads (GE Healthcare), washed 3× with wash buffer (10 mM Tris, pH 8.0, 110 mM KOAc, 2 mM MgOAc, 0.1% Tween-20), incubated with 200 µg CN_A1–391/B1–370_ cell extract with or without 200 µM competing peptide, and finally washed 3× with wash buffer (containing competing peptides if present in the previous step). For GST-CNA1 experiments involving S-A238L_157–239_, 20 µg cell extracts containing co-expressed GST-CNA1 and CNB1 were bound to beads as described above, then incubated with 350 ng purified S-A238L_157–239_ in the presence or absence of competing peptide. In both experiments, bound proteins were eluted by boiling samples in Laemmli buffer. Peptides for competition assays were synthesized by the Tufts University Core Facility. The amino acid sequences were: LxVPc1, DQYLAVPQHPYQWAK; LxVPmut, DQYAAAAQHPYQWAK; PVIVIT, GPHPVIVITGPHEE; and PVIVITscrambled, GPIVPIHVTHPGEE.

### Accession Numbers

The structure factors and coordinates for the CN-A238L complex have been deposited with the Protein Databank with accession number 4F0Z.

## Supporting Information

Figure S1 A238L binds CN via a PxIxIT and LxVP motif. (A) Recombinant S-tagged A238L_157–239_ incubated with GST-CNA and CNB co-purifies with GST-CNA (lane 5). Incubation with excess peptides encoding the LxVP site from NFATc1 (lane 1) or the high-affinity PxIxIT peptide PVIVIT (lane 3) interferes with A238L-CNA binding. Control peptides (lanes 2 and 4) do not interfere with binding. (B) Plot of CN rate as a function of A238L_200–239_ concentration at different RII concentrations ranging from 50–1,000 µM. Curve fit obtained by nonlinear regression using the Morrison equation to account for tight binding inhibition. Error bars indicate one s.d. from three independent experiments.
(TIF)

Figure S2 Role of the PxIxIT and LxVP sites in the CN-A238L interaction. Raw isothermal titration calorimetry data (upper panels) and derived binding isotherm plotted versus the molar ratio of titrant fit using a one-site model (lower panels) for CN_A1–391/B1–170_ titrated with: (A) WT A238L, (B) A238L PxIxIT mutant (PKIIIT mutated to AKAIAA), and (C) A238L LxVP mutant (FLCVK mutated to AACAA). Thermodynamic data and *K*_D_ values are summarized in Table 1.
(TIF)

Figure S3 A238L-CN polar interactions. (A) Stereo-view of the FLCVK_A238L_ interface. CN residues participating in the interaction are shown as grey (CNA) or beige (CNB) sticks, with A238L residues shown in purple. The multiple intra- and intermolecular hydrogen bonds that stabilize the A238L kink are shown as black dotted lines. (B) A238L residues immediately C-terminal to the ^206^PKIIIT^211^ motif, ^212^GCEDNVY^218^, are illustrated as sticks and labeled. CNA residues that interact with these A238L residues are also shown as sticks (black). Hydrogen bonds/salt bridge interactions are indicated by black dashed lines.
(TIF)

Figure S4 A238L LxVP motif mutant weakly inhibits RII dephosphorylation. (A) Dose-response plot of CN rate as a function of A238L_FLCVKmut_ at different RII concentrations ranging from 50–1,000 µM. Curves were fit by nonlinear regression using the Morrison equation. Error bars indicate one s.d. from three independent experiments. (B) Plot of CN rate as a function of [RII]. Data fit the Michaelis-Menten model for competitive inhibition. Points represent averages ± s.d. from three independent experiments. Concentrations of A238L_FLCVKmut_ are indicated.
(TIF)

Figure S5 Stereoview of the A238L electron density. (A) Sigma 2m*F_o_*-D*F_c_* electron density map of A238L contoured at 1σ to 1.70 Å (blue mesh). A238L shown as magenta sticks with the PxIxIT and LxVP motifs in green. (B) Close-up stereoview of the A238L LxVP motif, with LxVP residues labeled.
(TIF)

Table S1 Data collection and refinement statistics.
(DOCX)

Text S1 Supplementary materials and methods.
(DOCX)

## Abbreviations

AID: auto-inhibitory domain
CN: Calcineurin
CNA: calcineurin catalytic A subunit
CNB: calcineurin regulatory B subunit
CSA: cyclosporin A
DFT: density functional theory
ITC: isothermal titration calorimetry
MD: Molecular Dynamics
NFAT: nuclear factor of activated T-cells
pNpp: p-nitrophenyl phosphate
PP1: Protein Phosphatase 1
PP2B: Protein Phosphatase 2B
PP3: Protein Phosphatase 3
PPP: phosphoprotein phosphatase
RII: RII regulatory subunit of PKA
