# Supplementary figures and images for: The Molecular Mechanism of Substrate Engagement and Immunosuppressant Inhibition of Calcineurin
Source: PLoS Biol. 2013 Feb 26;11(2):e1001492. doi: 10.1371/journal.pbio.1001492 (PMC3582496; doi:10.1371/journal.pbio.1001492)

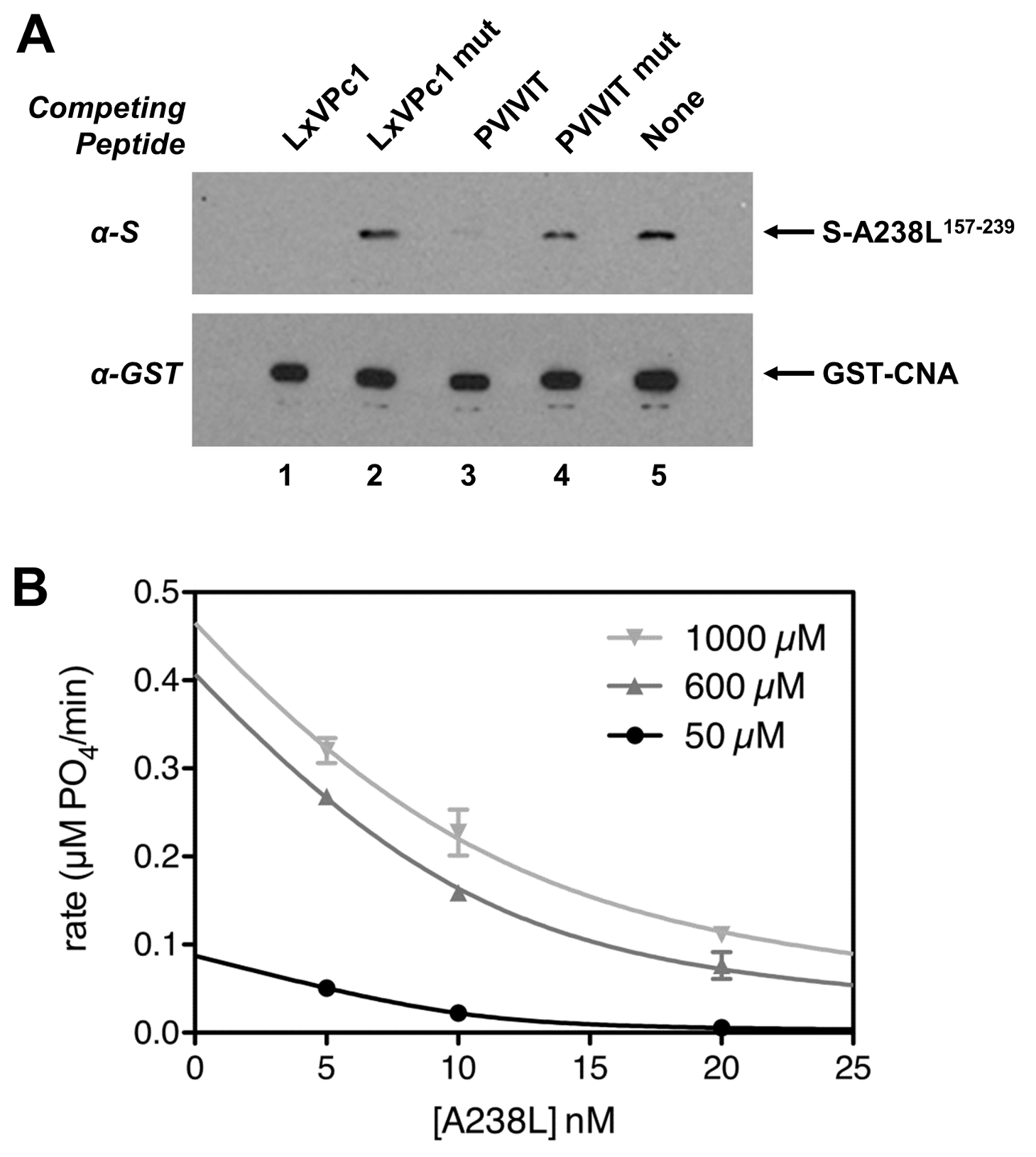

Supplement: Figure S1 — A238L binds CN via a PxIxIT and LxVP motif. (A) Recombinant S-tagged A238L157–239 incubated with GST-CNA and CNB co-purifies with GST-CNA (lane 5). Incubation with excess peptides encoding the LxVP site from NFATc1 (lane 1) or the high-affinity PxIxIT peptide PVIVIT (lane 3) interferes with A238L-CNA binding. Control peptides (lanes 2 and 4) do not interfere with binding. (B) Plot of CN rate as a function of A238L200–239 concentration at different RII concentrations ranging from 50–1,000 µM. Curve fit obtained by nonlinear regression using the Morrison equation to account for tight binding inhibition. Error bars indicate one s.d. from three independent experiments. (TIF) [file pbio.1001492.s001.tif]

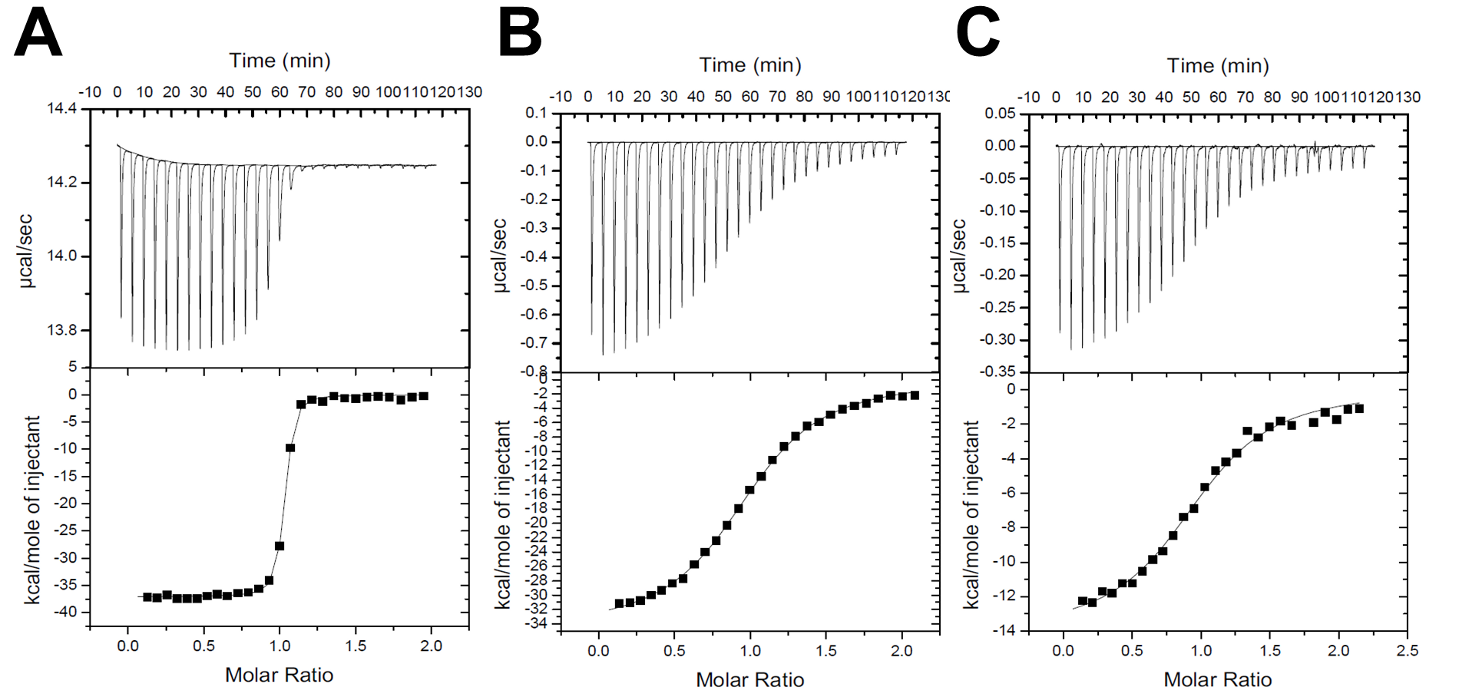

Supplement: Figure S2 — Role of the PxIxIT and LxVP sites in the CN-A238L interaction. Raw isothermal titration calorimetry data (upper panels) and derived binding isotherm plotted versus the molar ratio of titrant fit using a one-site model (lower panels) for CNA1–391/B1–170 titrated with: (A) WT A238L, (B) A238L PxIxIT mutant (PKIIIT mutated to AKAIAA), and (C) A238L LxVP mutant (FLCVK mutated to AACAA). Thermodynamic data and K D values are summarized in Table 1. (TIF) [file pbio.1001492.s002.tif]

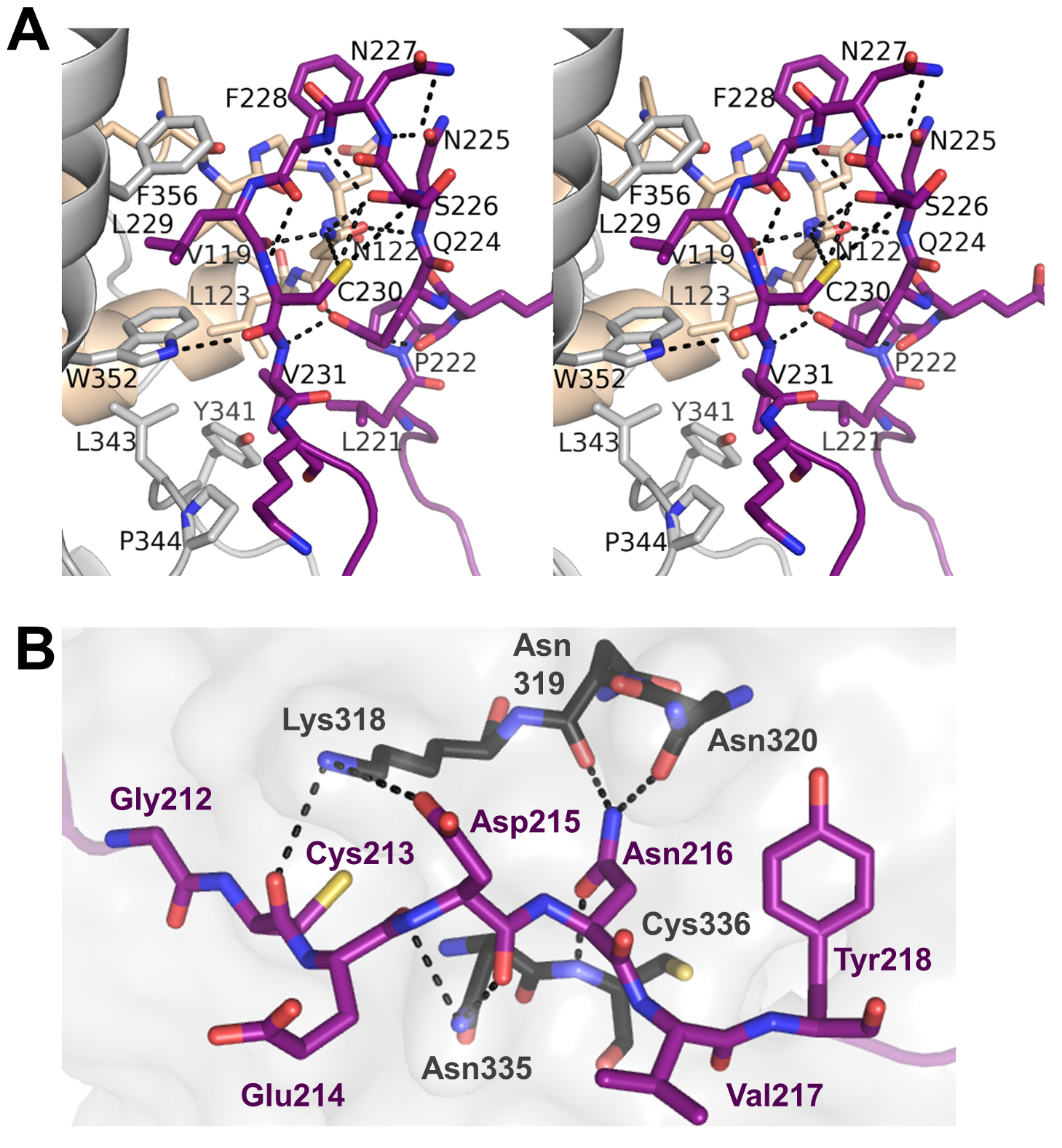

Supplement: Figure S3 — A238L-CN polar interactions. (A) Stereo-view of the FLCVKA238L interface. CN residues participating in the interaction are shown as grey (CNA) or beige (CNB) sticks, with A238L residues shown in purple. The multiple intra- and intermolecular hydrogen bonds that stabilize the A238L kink are shown as black dotted lines. (B) A238L residues immediately C-terminal to the 206PKIIIT211 motif, 212GCEDNVY218, are illustrated as sticks and labeled. CNA residues that interact with these A238L residues are also shown as sticks (black). Hydrogen bonds/salt bridge interactions are indicated by black dashed lines. (TIF) [file pbio.1001492.s003.tif]

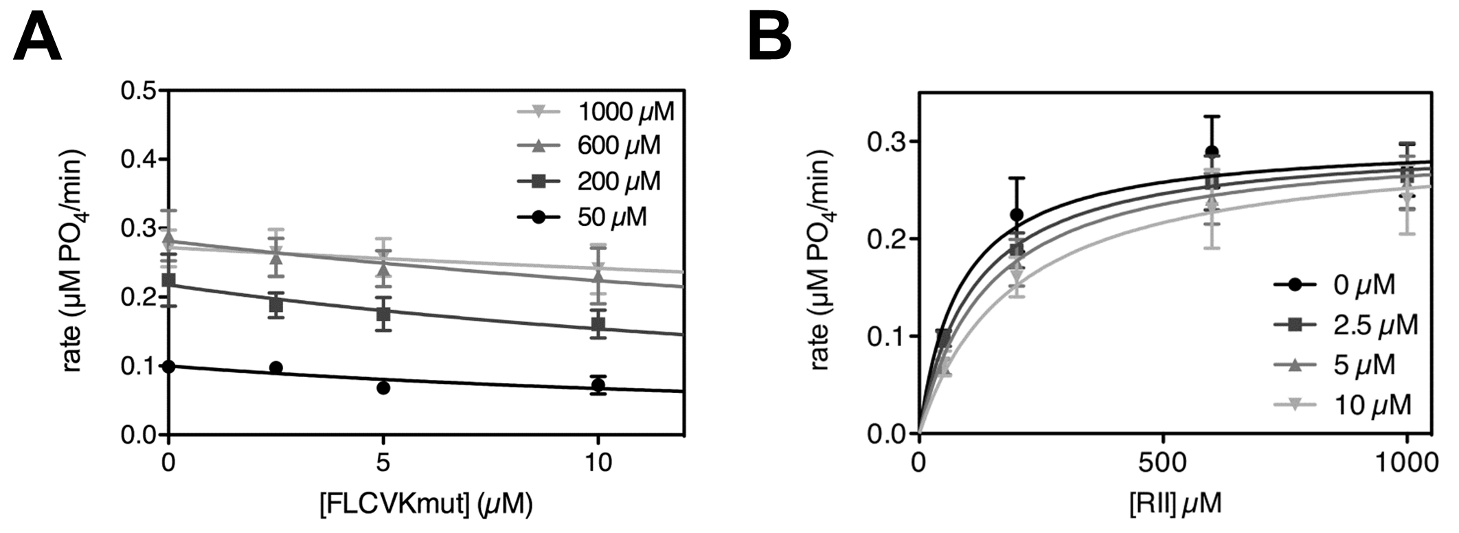

Supplement: Figure S4 — A238L LxVP motif mutant weakly inhibits RII dephosphorylation. (A) Dose-response plot of CN rate as a function of A238LFLCVKmut at different RII concentrations ranging from 50–1,000 µM. Curves were fit by nonlinear regression using the Morrison equation. Error bars indicate one s.d. from three independent experiments. (B) Plot of CN rate as a function of [RII]. Data fit the Michaelis-Menten model for competitive inhibition. Points represent averages ± s.d. from three independent experiments. Concentrations of A238LFLCVKmut are indicated. (TIF) [file pbio.1001492.s004.tif]

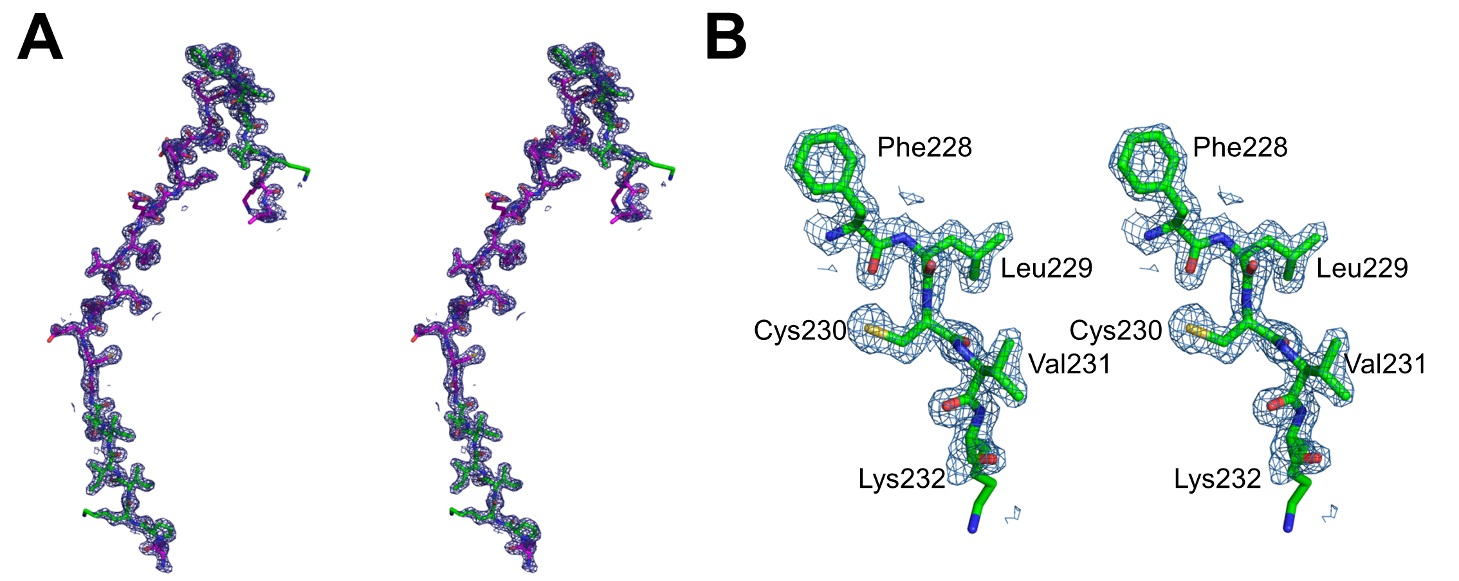

Supplement: Figure S5 — Stereoview of the A238L electron density. (A) Sigma 2mFo-DFc electron density map of A238L contoured at 1σ to 1.70 Å (blue mesh). A238L shown as magenta sticks with the PxIxIT and LxVP motifs in green. (B) Close-up stereoview of the A238L LxVP motif, with LxVP residues labeled. (TIF) [file pbio.1001492.s005.tif]
