# Supplementary material for: The Molecular Mechanism of Substrate Engagement and Immunosuppressant Inhibition of Calcineurin
Source: PLoS Biol. 2013 Feb 26;11(2):e1001492. doi: 10.1371/journal.pbio.1001492 (PMC3582496; doi:10.1371/journal.pbio.1001492)
Supplement: Table S1 — Data collection and refinement statistics. (DOCX) [file pbio.1001492.s006.docx]

**Table S1** **Data collection and refinement statistics**

|  | CN-A238L* |  |
| --- | --- | --- |
| **Data collection** |  |  |
| Space group | P2_1_ |  |
| Cell dimensions |  |  |
| *a*, *b*, *c* (Å) | 72.69, 48.98, 82.44 |  |
| β (°) | 104.4 |  |
| Resolution (Å) | 50.0-1.7 (1.73 – 1.70)** |  |
| *R*_sym_ or *R*_merge_ | 6.8 (29.9) |  |
| *I* / σ*I* | 20.4 (3.0) |  |
| Completeness (%) | 97.4 (87.5) |  |
| Redundancy | 3.0 (2.3) |  |
|  |  |  |
| **Refinement** |  |  |
| Resolution (Å) | 47.3 – 1.7 |  |
| No. reflections | 59661 |  |
| *R*_work_ / *R*_free_ | 15.8/17.8 |  |
| No. atoms |  |  |
| Protein | 5025 |  |
| Ligand/ion | 52 |  |
| Water | 470 |  |
| *B*-factors |  |  |
| Protein | 20.6 |  |
| Ligand/ion | 36.0 |  |
| Water | 32.6 |  |
| R.m.s. deviations |  |  |
| Bond lengths (Å) | 0.008 |  |
| Bond angles (°) | 1.016 |  |

*One crystal. **Values in parentheses are for highest-resolution shell.
