# Supplementary material for: The Molecular Mechanism of Substrate Engagement and Immunosuppressant Inhibition of Calcineurin
Source: PLoS Biol. 2013 Feb 26;11(2):e1001492. doi: 10.1371/journal.pbio.1001492 (PMC3582496; doi:10.1371/journal.pbio.1001492)
Supplement: Text S1 — Supplementary materials and methods. (DOCX) [file pbio.1001492.s007.docx]

**SUPPORTING INFORMATION FOR:**

**The structure of calcineurin with viral A238L reveals an inhibitory mechanism in common with immunosuppressants**

Simina Grigoriu^2^, Rachel Bond, Pilar Cossio, Jennifer A. Chen, Nina Ly, Gerhard Hummer, Rebecca Page, Martha S. Cyert* and Wolfgang Peti*

**Correspondence should be addressed to M.S.C (mcyert@stanford.edu) or W.P. (wolfgang_peti@brown.edu).*

**SUPPORTING PROTOCOLS**

**Cloning.** Cloning was carried out using standard protocols. The A238L_200-239_ sequence from the Malawi LIL20-1 strain was PCR-amplified and cloned into the bacterial expression vector RP1B [[1](#_ENREF_1)], which encodes an N-terminal Thio_6_- and His_6_-tag followed by a tobacco etch virus (TEV) protease cleavage site. The CN_A1-391/B1-170_ and the CN_A1-370/B1-170_ sequences were PCR-amplified and the open reading frames of both CNA and CNB subunits were cloned into the pET15b-derived p11 bicistronic bacterial expression vector (PSI:Biology-Materials Repository) as a single cassette, which contains an N-terminal His_6_-tag followed by a TEV protease cleavage site. A238L_200-239_ mutants were generated by direct cloning of annealed double-stranded overhang oligonucleotides into the digested A238L_200-239_ expression plasmid. GST-peptide fusion expression vectors were generated by the same method using pGEX4T3 (GE Life Sciences) as the parent vector. The A238L C-terminal domain truncation series was generated by PCR amplification of desired sequences and cloning into the yeast expression vector pRD56. A pET32a vector containing S-tagged A238L_157-239_ was generously provided by Linda Dixon (Pirbright Laboratory, UK). GST-tagged yeast CNA1_1-417_ (BJP3003) and CNB1-pET9a have been previously described [[2](#_ENREF_2)]. The mammalian expression vector pCDNASV5A238L encoding full-length A238L was provided by Linda Dixon. Docking motif mutants A238L_PKIIITmut_ and A238L_FLCVKmut_ were generated by site-directed mutagenesis.

**Protein expression and purification.** RP1B expression plasmid containing either wild-type or mutated (PKIIIT mutated to AKAIAA or FLCVK mutated to AACAA) A238L_200-239_ was transformed into BL21-(DE3) RIL *E. coli* cells (Agilent) and expression was carried out in Luria broth medium containing chloramphenicol (34 µg/ml) and kanamycin (50 µg/ml). Cell cultures were grown at 37°C under vigorous shaking (250 rpm) to an OD_600_ of 0.7. Cells were cooled at 4°C for one hour, while the shaker temperature was lowered to 18°C. Expression of wild-type or mutant A238L_200-239_ was induced by addition of 1 mM IPTG, and the cultures were grown for an additional 18 h at 18°C (250 rpm). The cells were harvested by centrifugation (6000x*g*, 15 min, 4°C) and stored at -80°C. The GST-peptide fusion plasmids and the p11 expression plasmid containing CN was transformed into BL21-(DE3) RIL *E. coli* cells (Agilent) and expression was carried out as described above for the A238L_200-239_ samples. For GST-CNA1_1-417_, CNB1, and S-A238L_157-239_, plasmids were transformed into BL21-(DE3) *E. coli* cells and expressed as described above.

Wild-type and mutant (PKIIIT🡪AKAIAA and FLCVK🡪AACAA) A238L_200-239_ were purified as follows. *E. coli* cells were resuspended in lysis buffer (50 mM Tris pH 8.0, 500 mM NaCl, 5 mM imidazole, 0.1% Triton X-100, EDTA-free protease inhibitor tablets [Roche]) and lysed by high-pressure cell homogenization (Avestin C3 Emulsiflex). The cell debris was removed by centrifugation (35000x*g*, 50 min, 4°C) and the supernatant was filtered and loaded onto a HisTrap HP column (GE Healthcare) equilibrated with 50 mM Tris pH 8.0, 500 mM NaCl, 5 mM imidazole. His_6_-tagged A238L_200-239_ was eluted from the column with a 5-500 mM imidazole gradient over 60 minutes. Fractions containing purified A238L_200-239_ were pooled, TEV (His_6_-tagged; produced in-house) was added for His_6_-tag cleavage, and samples were dialyzed against protein buffer (20 mM Tris pH 8, 500 mM NaCl) at 4°C for 16 h. The enzymatically-cleaved His_6_-tag and TEV protease were removed by Ni^2+^-affinity subtraction purification. A238L_200-239_ used for crystallographic complex formation was further purified by size exclusion chromatography (SEC; Superdex 75 26/60 [GE Healthcare]) equilibrated in 20 mM Tris pH 7.5, 500 mM NaCl, 0.5 mM TCEP. A238L_200-239_ was concentrated to ~1 mM and frozen at -80°C for later use. Finally, wild-type or mutant A238L_200-239_ used for ITC was concentrated to >1 mM immediately following the subtraction purification step, and stored at -80°C. Immediately prior to ITC experiments, A238L_200-239_ samples were thawed and SEC-purified in ITC buffer (20 mM Tris pH 7.5, 150 mM NaCl, 0.5 mM TCEP, 1.5 mM CaCl_2_). A238L_157-239_ was purified as described above up to and including the Ni^2+^- affinity purification step, and stored at -80°C.

His_6_-tagged CN_A1-391/B1-170_ used for ITC was purified over a HisTrap HP affinity column as described above for A238L_200-239_. Following removal of the His_6_-tag and TEV by subtraction purification, CN _A1-391/B1-170_ was further purified for ITC by SEC (Superdex 200 26/60 [GE Healthcare]) equilibrated in ITC buffer.

**CN-dependent gene expression assays in yeast.**GST or GST fused to full length or C-terminal truncations of A238L were expressed under control of the GAL1 promoter in *S. cerevisiae* strain SGY104 (*MATa lys2-801 ade2-101 trp1-Δ63 his3−Δ200 leu2-Δ1 ura3-52::TRP1-*2X CDRE*lacZ*), which harbors the CN-dependent reporter gene, 2X CDRE-lacZ [[3](#_ENREF_3)]. Cells were grown to mid-log phase with 4% raffinose as the carbon source, and expression was induced with 2% galactose and supplemented with 50 mM CaCl_2_ for 2.5 hrs to activate CN. β-galactosidase activity is reported as maximum rate OD_415_ change/minute/mg of protein and was determined as described in [[4](#_ENREF_4)] using 300 μg protein in each assay. Values represent an average of three independent extracts, each measured in triplicate. Error bars indicate the standard deviation.

**Luciferase reporter assays.** HEK293T cells were plated at 5 x 10^5^ cell/well in 24-well plates and co-transfected the next day with indicated amount of A238L, NFAT-Luc [[5](#_ENREF_5)], and Renilla-Luc^28^ DNA in 2 uL of Lipofectamine2000 (Invitrogen) in 100 uL of OptiMEM (Invitrogen). The ratio of A238L:NFAT-Luc:Renilla-Luc was as follows: 1: 2: 0.5, with the exception of A238L_PKIIITmut_ which was as follows: 5:2:0.5 to compensate for low expression. Fourteen hours after transfection, the cells were treated with 1 µM phorbol 12,13-dibutyrate (PDBu) (Sigma-Aldrich) to activate AP-1, and 1 µM ionomycin (Calbiochem). Luciferase levels were assayed 6 hours later using a homemade substitute [[6](#_ENREF_6)] and a Veritas 96-well luminometer (Turner Biosystems) according to the instructions of the manufacturer. The luminescence of each sample was measured by integrating for 2 s after injection of the luciferase substrate. NFAT-Luc activity was normalized to Renilla-Luc activity for analysis. Protein expression levels were verified by western blot with monoclonal anti-simian virus 5 (SV5) PK tag (Serotec) and monoclonal anti-GAPDH (Santa Cruz Biotechnology).

**SUPPORTING REFERENCES**

1. Peti W, Page R (2007) Strategies to maximize heterologous protein expression in Escherichia coli with minimal cost. Protein Expr Purif 51: 1-10.

2. Roy J, Li H, Hogan PG, Cyert MS (2007) A conserved docking site modulates substrate affinity for calcineurin, signaling output, and in vivo function. Mol Cell 25: 889-901.

3. Stathopoulos AM, Cyert MS (1997) Calcineurin acts through the CRZ1/TCN1-encoded transcription factor to regulate gene expression in yeast. Genes Dev 11: 3432-3444.

4. Bultynck G, Heath VL, Majeed AP, Galan JM, Haguenauer-Tsapis R, et al. (2006) Slm1 and slm2 are novel substrates of the calcineurin phosphatase required for heat stress-induced endocytosis of the yeast uracil permease. Mol Cell Biol 26: 4729-4745.

5. Park CY, Hoover PJ, Mullins FM, Bachhawat P, Covington ED, et al. (2009) STIM1 clusters and activates CRAC channels via direct binding of a cytosolic domain to Orai1. Cell 136: 876-890.

6. Dyer BW, Ferrer FA, Klinedinst DK, Rodriguez R (2000) A noncommercial dual luciferase enzyme assay system for reporter gene analysis. Anal Biochem 282: 158-161.
